# Superior bone fixation of conical compared with hemispherical trapezial cup design: an experimental radiostereometry study

**DOI:** 10.1186/s40634-023-00692-y

**Published:** 2023-11-30

**Authors:** Maiken Stilling, Lene Dremstrup, Torben Bæk Hansen, Janni Kjærgaard Thillemann

**Affiliations:** 1 Department of Orthopaedic Surgery, Aarhus University Hospital, Palle Juul-Jensens Boulevard 165 J801, DK- 8200 Aarhus N, Denmark; 2 Department of Clinical Medicine, Aarhus University, Aarhus N, Denmark; 3 AutoRSA Research Group, Orthopaedic Research Unit, Aarhus University Hospital, Aarhus N, Denmark; 4 Department of Orthopaedics, University Clinic for Hand, Hip and Knee Surgery, Gødstrup Hospital, Herning, Denmark

**Keywords:** Trapeziometacarpal arthroplasty, Trapeziometacarpal osteoarthritis, Cup fixation, Radiostereometry, Conical cup, Hemispherical cup

## Abstract

**Purpose:**

The most used cup designs for trapeziometacarpal (TMC) arthroplasty are of hemispherical and conical geometrical shape. Using a validated pig bone model, we compared the bone fixation using radiostereometry (RSA).

**Methods:**

Twenty saddle-shaped pig forefoot bones were prepared with insertion of bone markers and reaming. Hemispherical Type T cups (Beznoska, Kladno, Czech Republic) (*N* = 10) and conical Moovis cups (Stryker, Pusignan, France) (*N* = 10) of 9-mm diameter were inserted press-fit. The bones were fixed in cement blocks for stability, and the cups were loaded in a motorized test stand. First, a *low-pressure cyclic load test* (0—150N) with 130 compression cycles was performed. Next, a *push-in test* of progressive loads with 50N increments (range: 150–900N) was applied until a visual change in cup position appeared. Cup migration was evaluated with RSA after every new load application. Cup failure was defined as total translation > 0.5 mm between two load applications.

**Results:**

Both cup types tolerated a compression load of 450 N without failure. Beyond this load level, the total translation cup migration of mean 0.20 mm (95% CI 0.11; 0.30) for the Type T group was higher than mean 0.10 mm (95% CI 0.06; 0.15) of the Moovis group (*p* = 0.046). The Hazard ratio for failure was 0.52 (95% CI 0.12; 2.17) (*p* = 0.37), indicating that the risk of failure was two-fold higher in the Type T group.

**Conclusion:**

We conclude that conical TMC cups have superior fixation as compared to hemispherical cups above a loading level of 450 N, which correspond to a 3.8 kg tip-pinch.

In a clinical perspective, based on the fixation strength of both cup types, it seems safe to allow light-load activities of daily living such as buttoning a shirt and using a key shortly after surgery and until sufficient osseointegration is achieved.

## Background

Trapeziometacarpal arthroplasty is a treatment option for trapeziometacarpal (TMC) joint osteoarthritis (OA), which preserves thumb length and protect against secondary metacarpophalangeal joint hyperextension [8]. Recent publications show that TMC arthroplasty reduces pain, improves grip and pinch strength, and improves function as measured by patient reported outcome measures [7, 9, 25]. The first trapezial cup designs had early and frequent failure related to aseptic loosening with up to 44% failure at two years follow-up [17, 30]. Several cup designs and fixation methods including cementation, screw-fixation, and press-fit fixation have been introduced. Conical and hemispherical geometry design with cementless fixation has been continued and five to ten years survival rates have improved to 90.8 – 96% for hemispherical cups (Arpe cup, Zimmer Biomet, Warsaw, IN, USA, and Maiä cup, Groupe Lépine, Genay Cedex, France) [1, 5, 7, 12, 19, 32] and to 95 – 100% for conical cups (Moovis cup, Stryker, Pusignan, France and Ivory cup, Stryker, Kalamazoo, MI, USA) [13, 20, 25, 26].

For conical cups, the surface area and cup diameter have been shown to affect the primary mechanical stability and load tolerance after press-fit fixation in bone [28]. Considering the same cup diameter of conical cups and hemispherical cups, the surface area is smaller for hemispherical cups [28]. Therefore, the postoperative load tolerance until achievement of secondary osseointegration is likely higher for conical cups. Primary mechanical cup fixation is important for the achievement of later osseointegration and implant survival [21, 22, 24]. Radiostereometry (RSA) is a precise imaging method for the measurement of implant migration, and early implant migration has a high predictive value for longer-term fixation of hip and knee arthroplasty [18, 22, 23, 33]. RSA is a validated method for evaluation of cup fixation in TMC arthroplasty [15], which has been used for evaluation of prospective cup migration in clinical studies [16, 27] as well as for evaluation of primary mechanical cup fixation in an experimental pig bone model [14, 28].

The purpose of this study was to compare RSA measured migration of a press-fit hemispherical and a conical-shaped TMC cup in a pig bone model.

## Methods

### Pig bone model

We used the saddle-shaped bone from the forefoot of five to six-month-old Danish Landrace pigs. A total of 20 forefeet were included and randomly allocated to hemispherical (Type T) or conical (Moovis) cup in a 1:1 ratio. The forefeet were scanned by dual-energy x-ray absorptiometry (DXA) in a posterior-anterior position and the BMD was measured in the central part of the saddle-shaped bone [4] and reported as g/cm^2^. Thereafter, the saddle-shaped bones were dissected from the pig's forefeet and prepared for RSA with six 1-mm tantalum beads in the subchondral bone by use of a bead-injector (Kulkanon, Wennberg Finmek, Gunnilse, Sweden).

### Implants

**Fig. 1:**
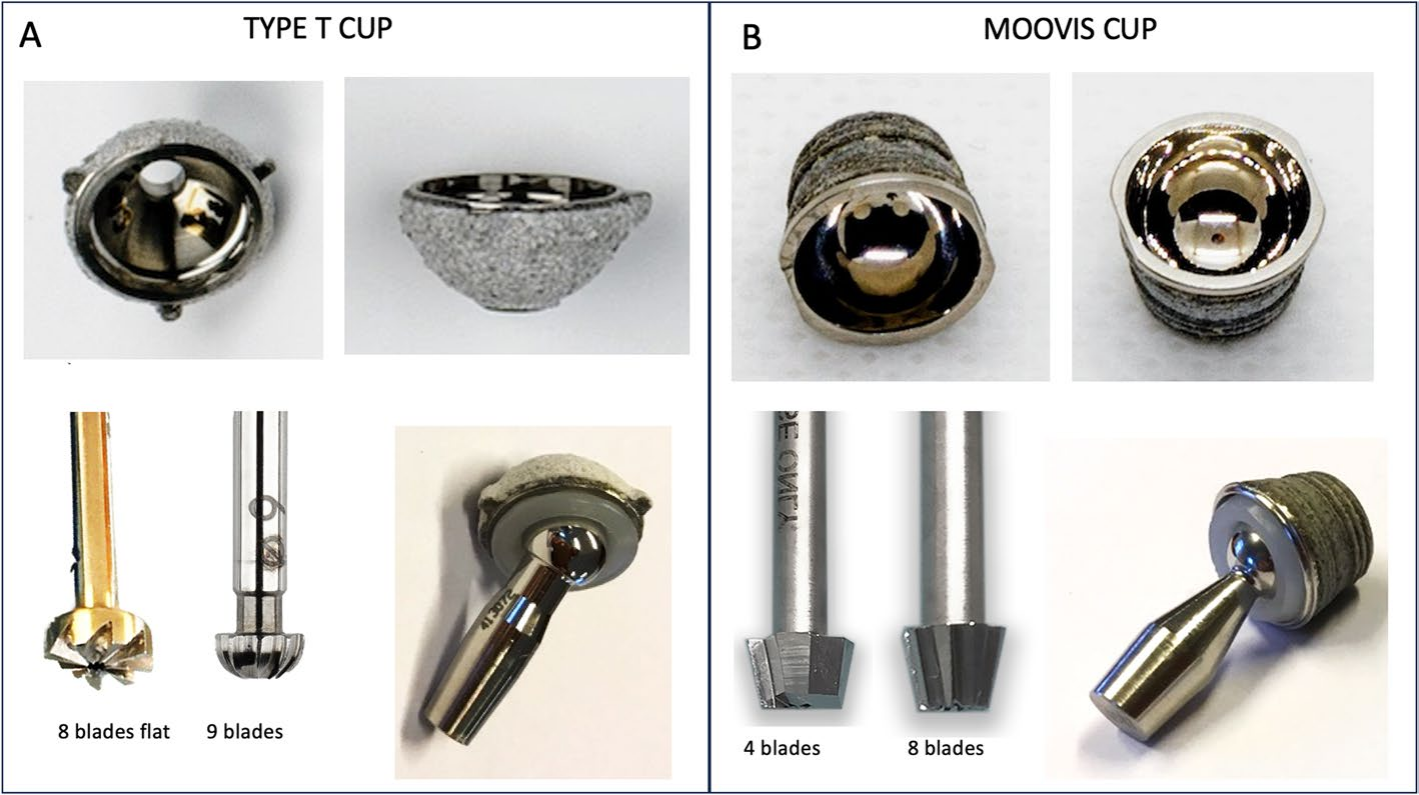
The design of **A** the hemispherical Type T cup and bone reamers and **B** the conical Moovis cup and bone reamers

The Type T cup (Beznoska, Kladno, Czech Republic) is a cementless press-fit hemispherical cup made of Ti6Al4V alloy (ISO 5832–3) with 3 rim-fins and an external diameter of 9 mm (Fig. 1A). The surface is coated with a plasma-spray double layer of titanium and hydroxyapatite. The titanium coating thickness is 0.1‐0.2 mm with a grain size of 0.075–0.18 mm and a roughness of 100–200 μm (international norm: ASTM F1580‐07). The hydroxyapatite coating of 2(Ca5(Po4)3OH) is 0.04–0.08 mm in thickness with a grain size of 0.05–015 mm and a roughness of 50–100 μm (international norm: ISO 13779‐1:2008). The inner surface with a Ø7.30 mm is manufactured by machine and polished by hand (roughness 0.2 Ra). The polyethylene liner is made of compression-molded and machined-manufactured modified crosslinked ultra-high-molecular-weight polyethylene (UHMWPE) (ISO 5834–2) and is fixed with a click-lock in the cup. The head-neck segment is made of molybdenum alloy (ISO 5832–12) and the head size is 5 mm in diameter. The original instrumentation (alignment reamers and cutting reamers) as well as the recommended surgical technique was used. The alignment reamer had eight cutterhead blades and the hemispherical size Ø8 and Ø9 mm reamers had nine cutterhead blades (Fig. 1A). The reaming system was designed for 0.1 mm under-reaming of the cup diameter. The full reamer depth was similar to the external depth of the cups.

The Moovis cup (Stryker, Pusignan, France) is a cementless press-fit conical cup CNC machined from chrome-cobalt alloy with external measures of 9 mm diameter and a dept of 7 mm (Fig. 1B). The surface is coated with a plasma-spray double layer of titanium and hydroxyapatite. The titanium coating is 0.1 mm and the hydroxyapatite coating is 0.1 mm in thickness. The dual-mobility polyethylene insert is manufactured from UHMWPE and has an external diameter of 8.2 mm and a dept of 6 mm. The head-neck segment is made of chrome-cobalt and the head size is 5 mm in diameter. The original instrumentation as well as the recommended surgical technique was used. There were two Ø9 mm cutting reamers (one with four cutterhead blades and one with eight cutterhead blades) intended for line-to-line reaming of the cup diameter (Fig. 1B). The full reamer depth was 1 mm more than the external depth of the hydroxyapatite coated area of the cups.

### Surgical technique

#### Type T cup

**Fig. 2:**
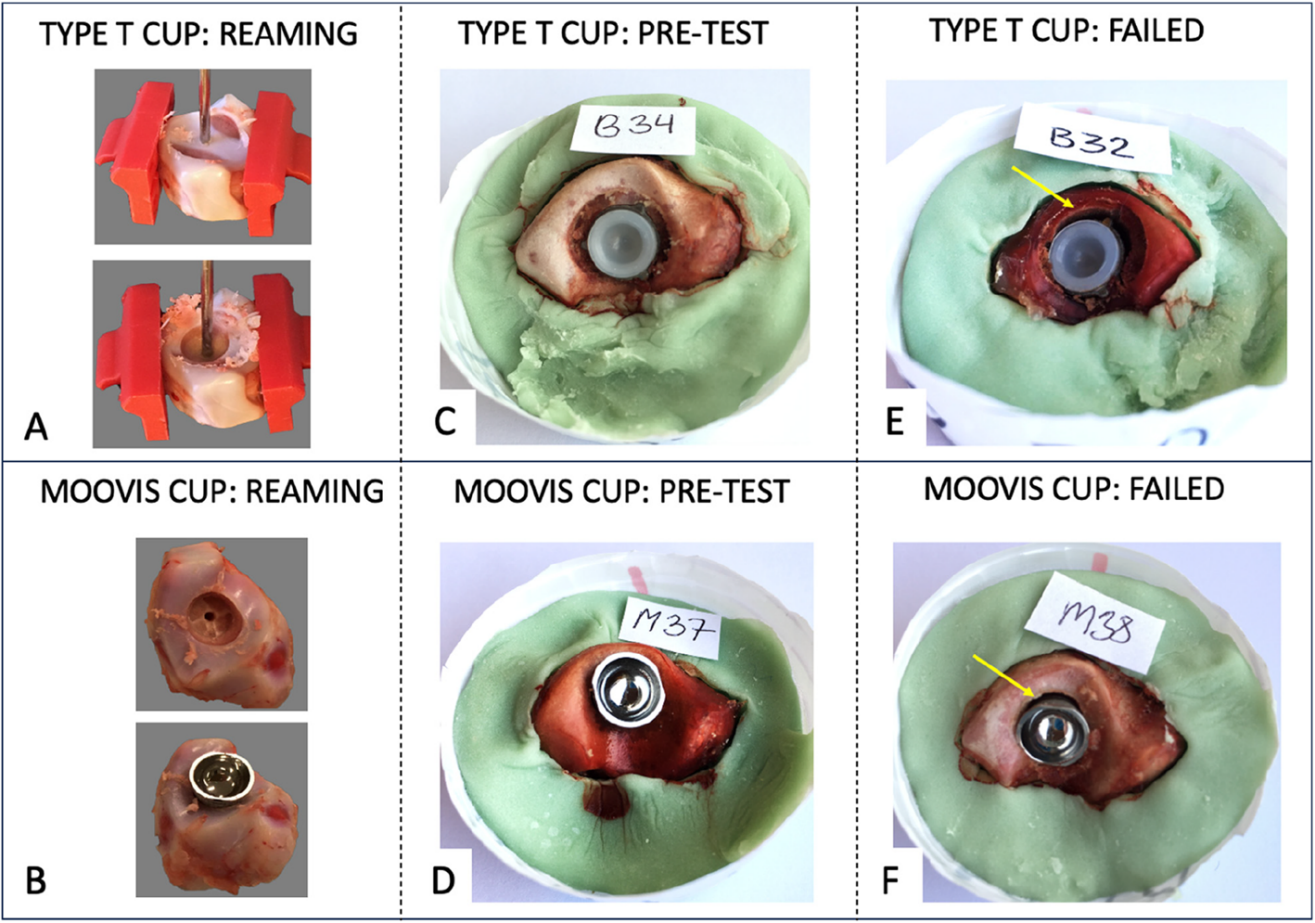
The **A** + **B** bone reaming, **C** + **D** press-fit cup insertion in the pig bone, and **E** + **F** failure with visual cup displacement of the Type 2 cup (upper panel) and the Moovis cup (lower panel)

A k-wire was inserted in the center of the articulate surface of the saddle-shaped bone. An alignment reamer was used to level the cartilage surface to the cortical bone (Fig. 2A). Cutting reamers size Ø8 mm and Ø9 mm on a reaming drill were used for preparation of the implant cavity. The cup was inserted over the guidewire using the original impactor and a hammer, and the cup was advanced to the cortical surface in order to achieve press-fit rim-fixation (Fig. 2A).

#### Moovis cup

A k-wire was inserted in the center of the articulate surface of the saddle-shaped bone. The two cutting reamers size Ø9 mm on a reaming drill were used for preparation of the implant cavity (Fig. 2B). The k-wire was removed and the cup was inserted using the original impactor and a hammer. The cup was advanced to the bottom of the implant cavity (until no further advancement of the implant was felt/seen during impaction) obtaining a tight cortical press-fit rim-fixation and leaving the non-coated rim of the cup outside the cartilage (Fig. 2B).

### Compression test

**Fig. 3:**
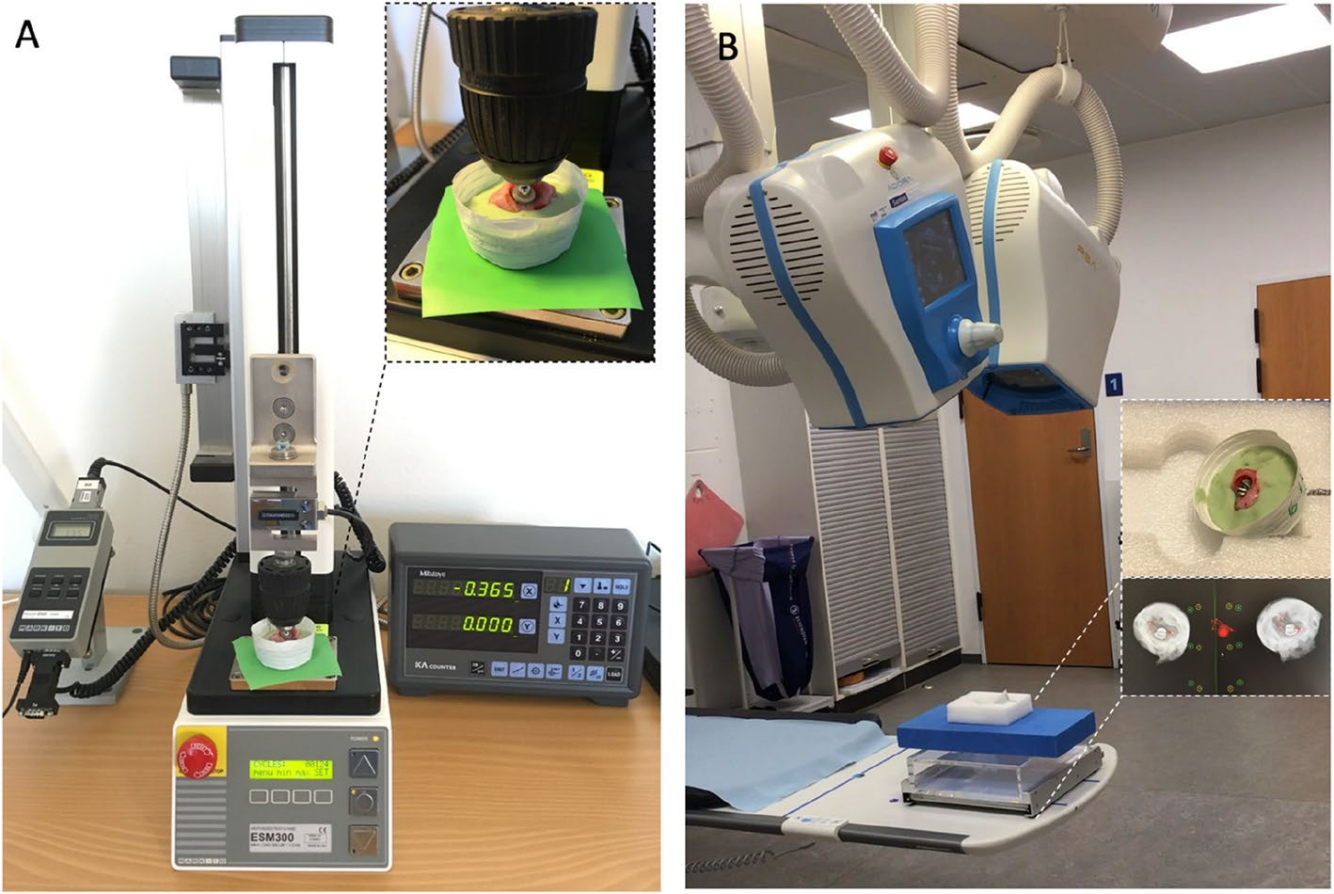
**A** The motorized Mark-10 test stand mounted with a 5 mm head-neck segment to apply a compression load on the cup during testing. **B** The DR RSA set-up with two ceiling-mounted x-ray tubes used for recording stereo-radiographs of the cup and bone-markers

**Fig. 4:**
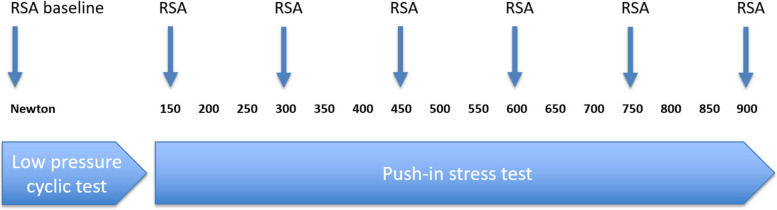
Overview of the two-phased study flow with a low-pressure cyclic load test (0-150N) and a push-in test stress test (150-900N) and regular RSA imaging

The set-up consisted of a Mark-10 motorized test stand ESM300 with a BGI SS200 controller (Swetest Instrument AB, Saltsjö-Boo, Sweden). A head-neck segment with a 5 mm head was mounted on the Mark-10 test stand and used to apply a compression load on the cup (Fig. 3A). For the Moovis cup, a dual-mobility polyethylene liner was mounted on the head-neck segment (Fig. 1B). For the Type T cup, the polyethylene liner was mounted in the cup (Fig. 1A). In this way, a centralized compression load of the cup into the saddle-shaped bone was performed. The load of the TMC joint was a combination of axial forces and horizontal forces [6]. In order to mimic the functional loading angle on the TMC cup in the natural TMC joint, the bones were rigidly fixed in separate blocks of bone cement (Heareus Palacos R + G) in a 20-degree angle on the horizontal plane (Fig. 2C + 2D) [14]. We performed two different compression tests on the cups (Fig. 4). First, a low-pressure cyclic load test (150N) was performed, which corresponds to a 15 kg load on the cup and a 1.25 kg tip-pinch. A force of 150N was not expected to result in any major force or migration of the cups, but the load simulated light-load repetitive activities of daily living [6, 29]. Second, a push-in test (150 – 900N) was performed, which corresponds to a max 92 kg load on the cup and a 7.6 kg tip-pinch [6]. A force of 900N was expected to challenge or exceed the primary mechanical cup fixation leading to cup migration or cup loosening [28].

In the *low-pressure cyclic load test* 130 compression cycles with load-increase from 0 – 150N was performed. The down speed was 4 mm/min and the dwell time was 4 s at the 150N load.

The *push-in test* applied 2 compression cycles of progressive load with 50N increments (range: 150–900N). The down-speed was 4 mm/min and the dwell time was 1 s between each load cycle. The tests were similar to an earlier study of different TMC implants [14].

### Radiostereometric Analysis (RSA)

Radiostereometric (RSA) imaging of the cups was performed at baseline, after the low-pressure cyclic load test, and repeated after every 150N load increment (300N, 450N, 600N, 750N, and 900N) (Fig. 4). The position of the cement-blocks with the bone-implant specimens was standardized in a radiolucent positioner at every RSA examination (Fig. 3B). The RSA set-up consisted of a direct digital stereo x-ray system (AdoraRSA suite; NRT, Aarhus, Denmark) with 2 ceiling-mounted x-ray tubes in vertical position with a 40-degree angulation between tubes (Fig. 3B). The source image distance (SID) between the tubes and detectors was 116 cm. Exposure settings were 66 kV, 200 mA, and 6.3 mAs. A wireless digital x-ray image detector (CXDI-801C; Canon, Tokyo, Japan) was slotted beneath a uniplanar Perspex hand calibration box (Medis Medical Imaging Systems BV, Leiden, the Netherlands). The resolution of the static images was 79 dpi. The RSA images were obtained as one DICOM file and converted to BMP images as the radiographs were split for RSA analysis.

### Model-based RSA analysis

Model-based RSA version 4.2 (RSAcore, LUMC, Leiden, the Netherlands) was used for analysis of the RSA images for measurement of cup migration. Each RSA image was calibrated, which aligned the bone-marker model in all examinations. Reverse engineered cup models were created by laser scanning of real implants of the same size (RSAcore, Leiden, the Netherlands). The cup model was fitted to the contour of the implant in the RSA images with a pose error of 0.99 mm (SD 0.01) for Type T cups and 0.69 mm (SD 0.01) for Moovis cups (Fig. 3B). The migration of the cup model with respect to a rigid marker-model of the tantalum beads in the bone was given by the software. The coordinate system of the two cup designs was similar with the y-axis placed in the central axis of the cup and pointing through the dome. The cup migrations were expressed as translations along the orthogonal x-, y-, and z-axes with respect to the marker-model and with reference to the baseline examination. The total translation (TT) was calculated as TT = √(x^2^ + y^2^ + z^2^). Rotations were not estimated due to the rotational symmetry of the Moovis cup. There were no left–right side issues to take into account.

### Radiostereometric precision

The RSA recordings were performed as double examinations for all cups at 150 N and at 750 N in order to evaluate precision. Precision was evaluated as cup migration between the double RSA recordings and reported as mean difference (systematic bias) with minimum and maximum values, and precision (SD of the mean differences × 1.96). Numbers for signed and absolute values were given.

### Sample size

The sample size was based on a clinical RSA precision study examining migration of the conical shaped threaded Elektra cup in patients [15]. Using a standard deviation of 0.32 mm, power of 80, and alpha of 0.05, we estimated a sample size of *n* = 8 per group for the detection of a mean difference in TT migration of 0.5 mm [16, 22].

### Data interpretation

Hansen and Stilling (2013) found a mean 0.19 mm TT with an upper 95% confidence interval of 0.5 mm for non-revised Electra cups in a clinical study. Based on these observations we defined a TT increase, between two compression tests, exceeding 0.5 mm as cup-loosening (failure). Cup migration along the x-, y-, and z-axes and TT until failure of the first cup (0 – 450N) was compared between groups, and cup migration until max load (0 – 900N) was displayed graphically. The maximum tolerated compression load (N) before the summed TT reached 0.5 mm, was assessed for all cups, compared between groups, and compared between failed and non-failed cups.

### Statistical analysis

The hypothesis of no difference in translation (primary outcome: TT, secondary outcomes: x, y, z axes) between groups before the first cup failure (0N to 450N) was analyzed using a univariate repeated measurement analysis (mixed model), with compression load (N) and cup type as fixed effects, and implant number as random effects. We used pairwise group comparisons for each compression load (N) to describe differences. Unequal standard deviations and correlations of the cup-type groups were considered in the analyses. Normal distribution of the mixed-model residuals was evaluated with quantile–quantile plots.

Using a student’s independent two sample t-test (equal variance) the BMD of the saddle-shaped pig bones and the TT of failed and non-failed cups, was compared between cup groups. The normality of continuous data was inspected using frequency and probability plots (quantile–quantile plots). Continuous data was reported as means with 95% confidence intervals (95% CI). Categorical data between groups was compared with a chi-squared test. The cumulative rate of cup survival was estimated using the Kaplan–Meier method. The level of significance was set at *p* < 0.05.

## Results

### Demographics

One Moovis cup was excluded from all analyses because of an error in the compression test. We did not experience any fractures of the saddle-shaped pig bones during reaming and cup insertion, or during the compression testing.

### Bone mineral density

The BMD was 0.43 g/cm^2^ (95% CI 0.38; 0.47) in the Type T group and 0.47 g/cm^2^ (95% CI 0.40; 0.53) in the Moovis group (*p* = 0.30).

### Cup migration

The *low-pressure cyclic loading test* (0-150N) resulted in TT cup migration of 0.07 mm (95% CI 0.04; 0.09) for the Type T group and of 0.07 mm (95% CI 0.02; 0.12) for the Moovis group (*p* = 0.95).

**Fig. 5:**
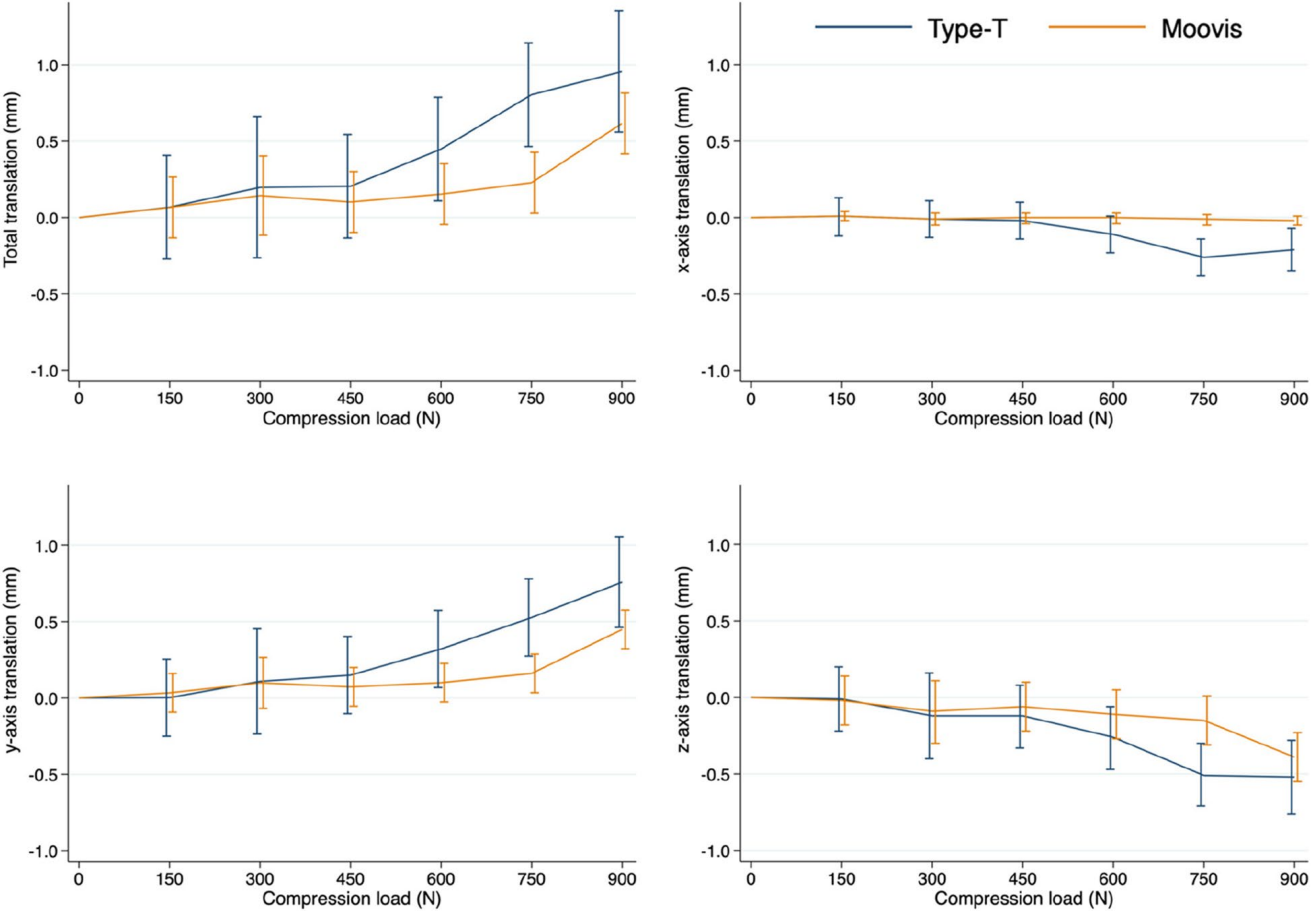
Cup migration evaluated with radiostereometry shown as Total translation (TT) and signed migration along the x, y, and z axes for the Type T and Moovis cup groups

During the *push-in test*, the first cup failures (defined as TT migration above 0.5 mm) between each compression test were seen at 600 N load in the Type T group and at 900 N load in the Moovis group. In the load-interval without failures (0-450N), TT cup migration was 0.20 mm (95% CI 0.11; 0.30) for the Type T group and 0.10 mm (95% CI 0.06; 0.15) for the Moovis group (*p* = 0.047). The signed cup translations (x, y, z) were similar between groups (*p* > 0.06) (Fig. 5).

At the final load (900N), 5 of 10 Type T cups and 3 of 9 Moovis cups had exceeded the migration failure limit of 0.5 mm TT between load tests (*p* = 0.46). The max TT translation for non-failed cups at 900N was 0.33 mm (95% CI 0.20; 0.46) for the Type T group and 0.23 mm (95% CI 0.09; 0.36) for the Moovis group (*p* = 0.27). The max TT translation for failed cups (TT > 0.5 mm between load tests) was 1.75 mm (95% CI 0.75; 2.75) for the Type T group and 1.44 mm (95% CI 0.65; 2.23) for the Moovis group (*p* = 0.56). Groups combined, the maximum TT migration was 0.24 mm (95% CI 0.11; 0.38) in the non-failure group (*n* = 11) and 1.28 mm (95% CI 0.88; 1.70) in the failure group (*n* = 8) (*p* = 0.0001).

### Migration pattern

Overall, the Type T group migrated more along all three orthogonal axes from 0 to 900N as compared with the Moovis group (Fig. 5). By visual assessment of the graphs, the Type T group migration started already at 450N, whereas the Moovis group was stable until 750N. Visually, this presented as a small cup tilt followed by subsidence and horizontal translation for the Type T cups. The Moovis cups had a similar migration pattern during failure (Fig. 5).

For the Type T group, the migration pattern for the whole loading test showed a maximum mean 0.26 mm (95% CI -0.38; -0.14) x-translation, 0.76 mm (95% CI 0.46; 1.06) y-translation, and -0.52 mm (95% CI -0.76; -0.28) z-translation. For the Moovis group, the migration pattern for the whole loading test showed a maximum mean -0.02 mm (95% CI -0.05; 0.01) x-translation, 0.45 mm (95% CI 0.32; 0.58) y-translation, and -0.39 mm (95% CI -0.55; -0.23) z-translation.

### Cup survival

**Fig. 6:**
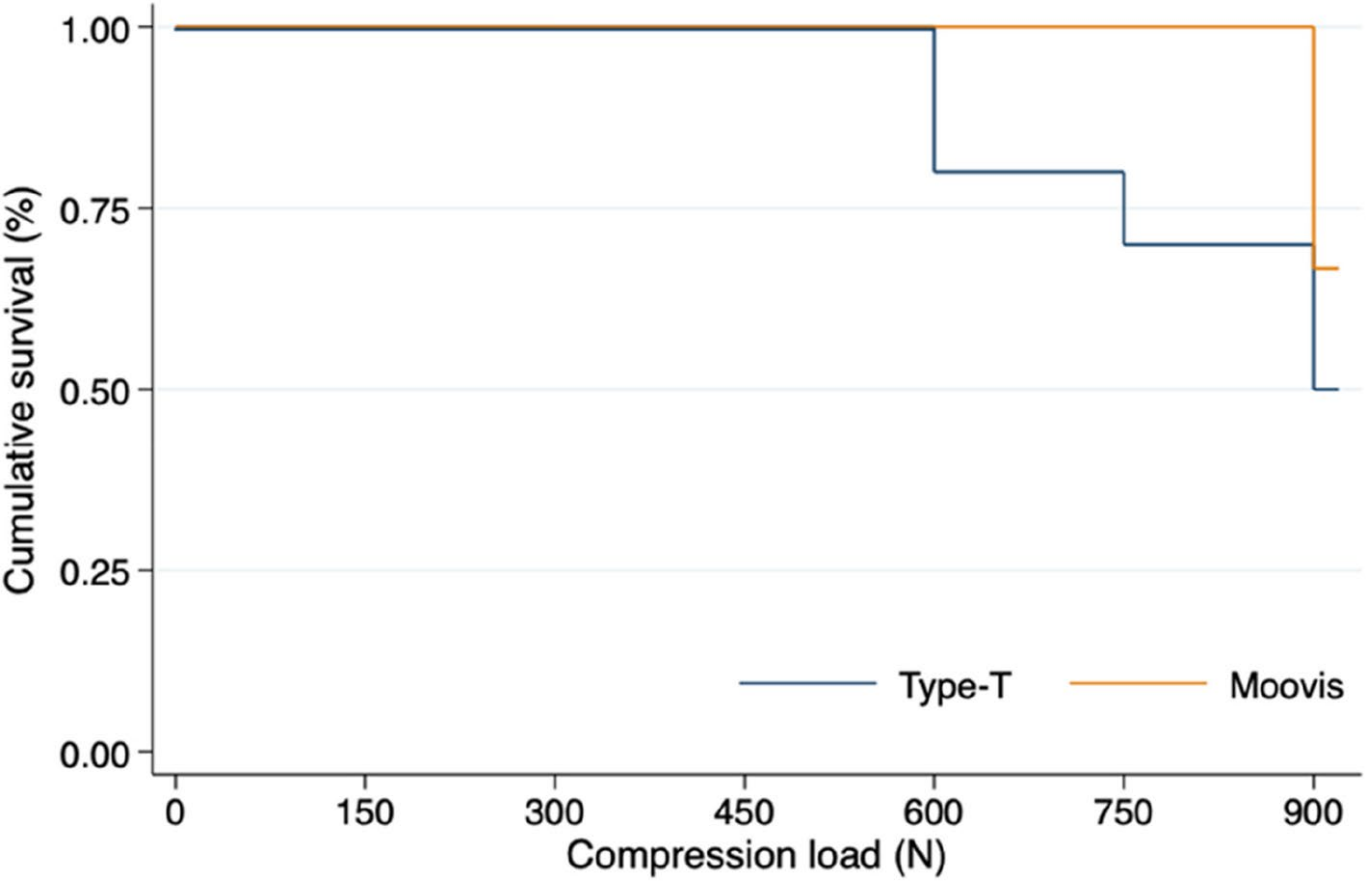
Kaplan–Meier cumulative survival plot for cup failure (TT migration > 0.5 mm)

The Kaplan–Meier cumulative survival estimate at 900 N, was 50% (95% CI 18; 75) in the Type T group and 67% (95% CI 28; 88) in the Moovis group (Fig. 6). The risk difference was 17% (95% CI -27; 60) and in favor of the Moovis group (*p* = 0.45). The Hazard ratio for failure was 0.52 (95% CI 0.12; 2.17) (*p* = 0.37), indicating that the risk of failure was two-fold higher in the Type T group.

### RSA precision

There was no systematic bias (mean difference) in TT and no difference in TT precision between the Type T group and the Moovis group (*p* > 0.71). The TT mean difference of the double examination stereo-radiographs was 0.031 mm and the precision was mean 0.066 mm (Table 1).

**Table 1:** Repeatability of cup migration as signed and absolute values from RSA double-examinations (*n* = 38 double examinations)

	Signed translations			Absolute translations			
	x	y	z	x	y	z	TT
Mean difference^a^	0.001	-0.001	-0.001	0.021	0.023	0.039	0.031
Precision^b^	0.053	0.062	0.105	0.032	0.042	0.072	0.066
Min. difference	-0.055	-0.100	-0.150	0.000	0.000	0.001	0.000
Max. difference	0.060	0.085	0.145	0.060	0.100	0.150	0.146

Numbers are reported as translation in mm

^a^mean difference between double examinations (systematic error)

^b^precision was calculated as 1.96 × SD (random error)

*TT* Total translation

## Discussion

The focus of this experimental study was to evaluate mechanical TMC cup fixation in the saddle shaped bone of a pig bone model. The study showed that beyond a compression load of 450 N the hemispherical Type T cup group had higher cup migration, and a two-fold higher risk of failure, as compared with the conical Moovis cup group.

There may be several explanations to these findings. The surface area of a 9 mm conical Moovis cup is 31% larger than for a 9 mm hemispherical cup. Thus, conical cups have better bone support at the time of primary cup fixation. Furthermore, for a larger cup diameter, the surface area of the Moovis cups has a greater percentage increase compared to the Type T cup. An experimental study of the conical Konos cup (Beznoska, Kladno, Czech Republic) inserted with cortical bone rim fixation showed that the risk difference for failure was 63% in favor of 10 mm cups compared to 9 mm cups [28].

The center of rotation in ball-and-socket cup designs defines the resultant force vector on the cup. For both the Moovis cup and the Type T cup the rotation center is level with the cup base. The hemispherical Type T cup is inserted level to the cortical bone surface, whereas the conical Moovis cup is designed to protrude slightly from the cortical bone surface. With a center of rotation outside the cortical bone surface the inclined resultant force vector increases in magnitude, which may cause eccentric loading and jeopardize mechanical bone fixation. Finite element analysis has shown that conical cups with a center of rotation at the bone surface deliver less stress to the cortical bone rim during angular loading of 30° compared with conical cups with a center of rotation that is above the bone surface [30]. The extrinsic center of rotation was probably the main cause of early failures with the Motec (Swemac AB, Linköbing, Sweden) cup design [30, 31].

In the present study, the TT before failure of the first cup (450N) was a mean of 0.20 mm for the Type T group and a mean of 0.10 mm for the Moovis group. In comparison, a recent similar experimental study of the conical Konos cup reported a TT of mean 0.23 mm at 450N [28].

A load of 450N resembles a hand grip of 45.9 kg and a tip-pinch of 3.8 kg [6]. In a 2-year follow-up of 200 patients operated with the Moovis cup, the hand grip for men and women combined was reported to mean 29 kg and none had grip strength above 45.9 kg [9]. In a population with osteoarthritis of the hand, the finger forces used during activities of daily living that primarily used the thumb and index finger in a precision grip were studied. The maximum force applied to the thumb ranged from 7.9 N (± 1.8) (approximately 1 kg) during a shirt button task to 30.7 N (± 3.7) (approximately 3.5 kg) during plug-in of a toaster [29]. Thus, a 450N load seems to be a clinically relevant upper limit for testing primary mechanical cup fixation.

Load- and shear-stresses at the implant-bone interface may not be the same with different cup designs. In the present study, the migration pattern on the graphical presentations of the 0 – 900N push-in test showed more subsidence of Type T cups at increasing loads as compared with Moovis cups. Furthermore, Type T cups translated horizontally to the articular bone surface along the z-axis indicating a failure pattern with loss of cortical rim-fixation and peri-prosthetic bone support. Visually, this presented as a cup-tilt followed by subsidence and horizontal translation. For the Moovis cups, the failure pattern was mainly subsidence with slight horizontal translation, but the migration leading to failure started at higher compression loads. The advantage of the Moovis cup design is that the geometry allows for some subsidence without loss of cortical rim-fixation. Perhaps, the cortical rim-fixation may even improve with loading of the conical Moovis cup during daily activities. The disadvantage of the conical cup shape is the risk of intra-operative stress fracture upon impaction of the cup [9]. Yet, there is also a risk of intra-operative fracture with press-fit hemispherical TMC cup designs [10, 12]. Intra-operative fracture as well as cup survival may be related to bone quality. In hip replacement surgery both stem and cup migration, as a predictor of implant survival, is higher in patients with low systemic bone mineral density (T-score < -1) [2, 11]. Bone antiresorptive treatment with RANK-L inhibitors (Denosumab) increase the periprosthetic bone mineral density in clinically relevant regions of the proximal femur, but the treatment response is not associated with a reduction in stem migration [3]. Coating roughness and porosity may also affect implants' primary mechanical fixation, and super-coatings such as hydroxyapatite have been optimized for years to aid osseointegration. However, in the present study, both cup types were coated with a similar plasma-spray double layer of titanium and hydroxyapatite.

The study was performed in a pig bone model and has natural limitations i.e., primary mechanical cup fixation cannot be directly translated to humans and cannot predict long-term cup fixation and survival. However, it benefits from a standardized setting utilizing a highly precise and validated quantitative imaging and analysis method. Further, the load application on the cups can be argued to have a high clinical relevance.

We conclude that conical TMC cups have superior fixation as compared with hemispherical cups above a loading level of 450 N, which corresponds to a 3.8 kg tip-pinch. In a clinical perspective, based on the fixation strength of both cup types, it seems safe to allow light-load activities of daily living such as buttoning a shirt and using a key shortly after surgery and until sufficient osseointegration is achieved.

## Data Availability

The datasets used and/or analyzed during the current study are available from the corresponding author on reasonable request.

## Abberviations

BMD: Bone mineral density
CNC: Computer Numerical Control
CI: Confidence interval
DXA: Dual-energy x-ray absorptiometry
OA: Osteoarthritis
RSA: Radiostereometry
TMC: Trapeziometacarpal
TT: Total translation
Type T: Hemispherical shaped cup type
Moovis: Conical shaped cup type
UHMWPE: Ultra-high-molecular-weight polyethylene
